# Enhanced dewaterability of waste activated sludge by a combined use of permanganate and peroxymonosulfate

**DOI:** 10.1039/c9ra03781k

**Published:** 2019-09-02

**Authors:** Lu Luo, Yongjian Ge, Shuyu Yuan, Yanghai Yu, Zhou Shi, Shiqing Zhou, Jing Deng

**Affiliations:** Key Laboratory of Building Safety and Energy Efficiency, Ministry of Education, Department of Water Engineering and Science, College of Civil Engineering, Hunan University Changsha Hunan 410082 P. R. China shiqingzhouwater@163.com; China United Engineering Corporation Limited Hangzhou 310052 P. R. China; College of Civil Engineering and Architecture, Zhejiang University of Technology Hangzhou 310014 P. R. China 1029877668@qq.com

## Abstract

Ever-increasing efforts have been made to develop rapid and practical conditioning methods of sludge dewatering. This study demonstrated an innovative combination of potassium permanganate (KMnO_4_) and peroxymonosulfate (PMS) for sludge dewatering. The combined use of KMnO_4_ and PMS (KMnO_4_/PMS) showed its superiority in improving sludge dewaterability over the separate use of KMnO_4_ or PMS. By dosing 4 mmol g^−1^ VSS KMnO_4_ and 3 mmol g^−1^ VSS PMS, the dewaterability of waste activated sludge (WAS) significantly enhanced as capillary suction time (CST) decreased from 73.65 s to 24.65 s while the water content of dewatered sludge cake (*W*_C_) decreased from 78.96% to 70.47%. Apart from CST and *W*_C_, the KMnO_4_/PMS process could also affect negative zeta potential, sludge flocs size and the concentrations of protein and polysaccharide in extracellular polymeric substances (EPS). The enhanced sludge dewaterability and changes of the physicochemical characteristics of the WAS samples during the KMnO_4_/PMS process were actually ascribed to sulfate radicals (SO_4_˙^−^) and hydroxyl radicals (HO˙) *in situ* generated *via* PMS activation by manganese oxides (MnO_*x*_) in the states of MnO_2_ and Mn_3_O_4_ transferred from KMnO_4_ oxidation, which was verified by transmission electron microscopy (TEM), energy dispersive X-ray spectroscopy (EDX) techniques and radical scavenging experiments. Moreover, the Fourier transform infrared spectroscopy (FTIR) analysis further confirmed that the *in situ* generated SO_4_˙^−^ and HO˙ could improve sludge dewaterability. Thus, the KMnO_4_/PMS process could be considered as a promising conditioning method of sludge dewatering.

## Abstract 2

Ever-increasing efforts have been made to develop rapid and practical conditioning methods of sludge dewatering.

## Graphical abstract

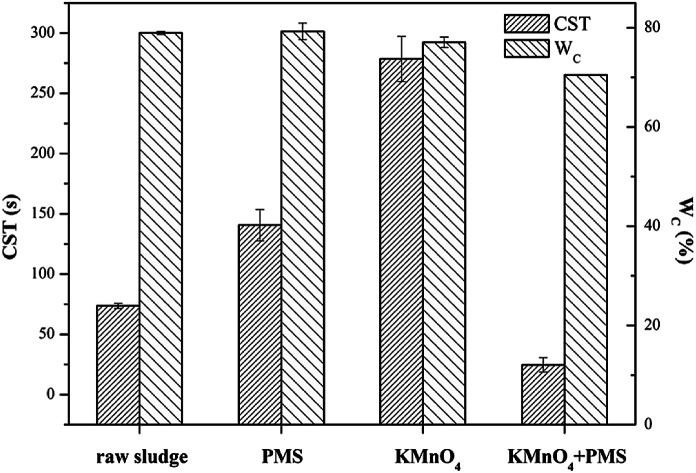

## 1. Introduction

The management of large quantities of waste activated sludge (WAS) produced from activated sludge processes has posed great challenges to municipal wastewater treatment plants (WWTPs) due to great economic and environmental burden.^1–4^ As reported, the treatment and disposal of excess sludge usually accounts for up to 60% of the total operating cost in WWTPs.^2^ Consequently, how to substantially cut down this kind of cost has become a significant issue, and many researchers and practitioners have tried their best to tackle this problem. WAS has a high water content in the form of free and bound water (over 90%).^5^ And sludge dewatering has been generally considered as a promising strategy in reducing sludge volume and cost of sludge transport and ultimate disposal.^3,4^ However, the sludge dewatering process is usually inhibited due to the presence of extracellular polymeric substances (EPS) that can retain a large amount of water, especially bound water.^6,7^ Hence, increasing efforts have been focused on development of efficient sludge dewatering techniques, which can promote the release of bound water from EPS. So far, thermal, ultrasonication, freezing and thawing, and chemical oxidation have been investigated to improve sludge dewaterability.^7–12^ Among all the developed methods, advanced oxidation processes (AOPs) have recently shown their superiority in sludge disintegration and dewatering.

AOPs usually rely on the produced highly reactive radical species, such as hydroxyl radicals (HO˙) and sulfate radicals (SO_4_˙^−^) to disrupt WAS and degrade EPS, thereby enhancing sludge dewaterability.^2,13,14^ It has been reported that the Fenton (combination of H_2_O_2_ and Fe^2+^) and Fenton-like (*e.g.*, simultaneous addition of H_2_O_2_ and Fe^3+^) processes based on HO˙ production could efficiently improve the dewaterability of WAS.^1,7^ However, only under strongly acidic conditions (pH = 2–5) can these processes generate sufficient amounts of HO˙ for better sludge dewaterability, which significantly hindered the sludge dewatering process and subsequent disposal.^15–17^ Compared to HO˙ (*E*^0^ = 1.8–2.7 V), SO_4_˙^−^ owns a higher redox potential (*E*^0^ = 2.5–3.1 V), a longer life time (30–40 μs) and a wider working pH range (pH 4–9).^4,18^ Therefore, pretty much attention has been recently paid to the SO_4_˙^−^-based AOPs as new alternative methods of sludge conditioning. SO_4_˙^−^ is usually generated through the activation of peroxydisulfate (PDS, S_2_O_8_^2−^) and peroxymonosulfate (PMS, HSO_5_^−^) by ultraviolet (UV), heat, base and (transition) metal catalysts.^19–25^

Our recent study investigated α-MnO_2_ with different morphologies (nanoparticles, nanoflowers and nanorods) synthesized *via* a facile hydrothermal method as activators of PMS for ciprofloxacin (CIP) degradation, and found that α-MnO_2_ nanoflowers achieved much higher degradation efficiency of CIP than the other three MnO_2_ (α-MnO_2_ nanorods, α-MnO_2_ nanoparticles and commercial MnO_2_), which would be attributed to its higher catalytic activity of PMS and larger quantities of SO_4_˙^−^ produced from its activation of PMS.^26^ Interestingly, Cui *et al.* recently studied chemical oxidation of benzene and trichloroethylene (TCE) by an innovative combined use of permanganate (KMnO_4_) and PMS (KMnO_4_/PMS), and also found that it was mainly colloidal and amorphous MnO_2_*in situ* generated from KMnO_4_ oxidation that activated PMS to trigger powerful SO_4_˙^−^-mediated oxidation of both benzene and TCE.^27^ As known to us all, both KMnO_4_ and PMS have been widely used as powerful oxidants to destruct organic pollutants in water and wastewater. Recently, the two powerful oxidants were also applied to enhance the filterability of waste activated sludge.^28,29^ Wu *et al.* reported that KMnO_4_ could efficiently disintegrate the excess sludge with soluble chemical oxygen demand (SCOD) increasing by 3473%, and that the optimal KMnO_4_/sludge solid mass ratio was 0.1 with a stable disintegration degree (DD_COD_) of about 34% while suitable reaction time was 30 min.^28^ Niu *et al.* found that PMS oxidation could effectively break sludge particles, and that EPS increased significantly and transferred to slime layer after PMS treatment.^29^ Yang *et al.* also reported that PMS could facilitate the disintegration of WAS and the biodegradability of organics could be enhanced after treatment by PMS.^30^ Although it has been proven that KMnO_4_ and PMS could disintegrate WAS and enhance sludge dewaterability, the synergistic effect of both oxidants on the dewaterability of WAS still remains unknown.

In this paper, we proposed a combined use of KMnO_4_ and PMS (KMnO_4_/PMS) for sludge dewatering. The dewaterability of WAS during the KMnO_4_/PMS process by the measurement of variations in capillary suction time (CST) and water content of dewatered sludge cake (*W*_C_) was initially investigated. Then, the physicochemical characteristics of WAS (CST, *W*_C_, zeta potential, particle size and the concentrations of protein and polysaccharide in EPS) at different dosages of KMnO_4_ and PMS were further studied. Finally, the mechanism of sludge dewatering by the KMnO_4_/PMS process was deeply explored.

## 2. Materials and methods

### 2.1. Raw sludge and chemicals

The raw sludge was collected from Qige municipal wastewater treatment plant in Hangzhou, China. A sieve with 20 mesh was then used to remove big gravels and debris from the collected sludge samples. The supernatant was finally decanted to acquire the denser sludge samples used in this study after plain sedimentation. The acquired denser sludge samples were stored at 4 °C in a refrigerator prior to use, and all the experiments were completed within 48 h. The basic characteristics of the sludge samples were measured and shown in Table 1.

**Table 1:** Basic characteristics of sludge samples

Parameter	Unit	Value
pH	—	6.85 ± 0.05
Water content	%	96.205 ± 0.005
Volatile suspended solids (VSS)	g L^−1^	30.364 ± 0.394
Total suspended solids (TSS)	g L^−1^	39.133 ± 0.951
VSS/TSS	%	77.355 ± 0.875
CST	s	73.65 ± 1.45

All the chemicals were used without further purification. Peroxymonosulfate (Oxone®, KHSO_5_·0.5 KHSO_4_·0.5 K_2_SO_4_, KHSO_5_ ≥47%) was provided by Aladdin Co., Ltd. (Shanghai, China). Permanganate (KMnO_4_, ≥99.5%), sodium hydroxide (NaOH, ≥96.0%), and sulfuric acid (H_2_SO_4_, 95.0–98.0%) were supplied by Sinopharm Chemical Reagent Co., Ltd. (Shanghai, China).

### 2.2. Experimental procedures

All the sludge dewatering experiments were conducted in an Erlenmeyer flask with a volume of 500 mL. In each test, 300 mL sludge sample was added and then mixed with a stoichiometric amount of PMS. When PMS was completely dissolved under thorough stirring, H_2_SO_4_ or NaOH was used to adjust the solution pH to 7. Finally, the reaction was immediately initiated by dosing a calculated amount of KMnO_4_ in a water bath apparatus (300 rpm), and was ceased after 120 min. All the experiments were duplicated at least, and the average values with standard deviations were presented.

### 2.3. Analytical methods

#### 2.3.1. CST and *W*_C_ determination

The dewaterability of WAS is commonly evaluated by CST and *W*_C_. CST was measured using a CST instrument (Type 304, Triton Ltd., UK) equipped with an 18 mm diameter funnel and a standard Whatman no. 17 chromatography-grade paper. To determine *W*_C_, the sludge samples were pretreated by a vacuum filtration method. Briefly, 50 mL sludge sample was poured into a standard Buchner funnel and filtered at a constant vacuum pressure for 15 min. The water content of the vacuum-filtered sludge cake trapped by the filter paper was measured according to the standard method, and calculated according to eqn (1):1
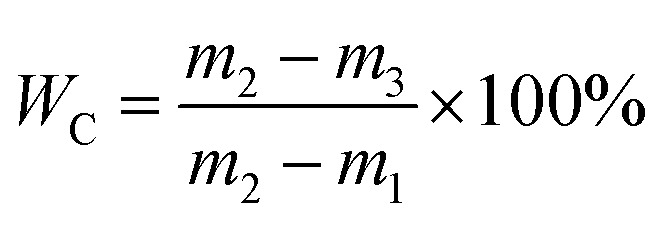
where *m*_1_ is the weight of the filter paper, *m*_2_ is the weight of the vacuum-filtered sludge cake, and *m*_3_ is the weight of the vacuum-filtered sludge cake completely dried at 105 °C.

#### 2.3.2. EPS extraction and analysis

EPS were extracted using a heat extraction method described by Yu *et al.*^6^ Briefly, 50 mL sludge sample was centrifuged at 4000*g* for 5 min at 4 °C to acquire the supernatant as soluble EPS (S-EPS). The sludge pellets were then diluted with a 0.05 w/v % NaCl solution that was pre-heated to 70 °C. The diluted sludge solution was immediately sheared by a vortex mixer for 1 min and centrifuged at 4000*g* for 10 min at 4 °C to collect the supernatant as the loosely bound EPS (LB-EPS). The residual sludge pellets were resuspended in a 0.05 w/v % NaCl solution and heated to 60 °C in a water bath for 30 min. At short notice, the resuspended sludge solution was centrifuged at 4000*g* for 15 min at 4 °C, and the supernatant was collected as the tightly bound EPS (TB-EPS).

Before the analyses of protein and polysaccharide (assumed as dominant components of EPS), the EPS samples were pretreated with an optimal amount of Na_2_SO_3_ to quench the residual colored KMnO_4_. The pretreated EPS sample was then filtered through a 0.45 μm cellulose acetate membrane. The protein content was quantified by the modified Lowry–Folin method using bovine serum albumin as the standard.^31^ The polysaccharide content was determined by the phenol-sulfuric acid method using glucose as the standard.^32^

#### 2.3.3. Other analyses

The zeta potential of the WAS samples was analyzed using a Zetasizer Nano (Nano-ZS90, Malvern Instrument Ltd., UK). The particle size distribution was measured by a laser particle size analyzer (LAP-W2000H) using ultrapure water as the dispersant. The Fourier transform infrared spectroscopy (FTIR) analysis of sludge samples was recorded in a wavenumber range of 4000–400 cm^−1^ by a Nicolet 6700 spectrometer (Thermo, USA).

Given the difficulty in separating the formed solids from the conditioned WAS samples, the extracted EPS solution was used and treated with the KMnO_4_/PMS process to monitor whether manganese oxides (MnO_*x*_) formed after KMnO_4_ oxidation of the organics in EPS. Briefly, after the extracted EPS solution was treated with the KMnO_4_/PMS process, the reaction solution was filtered through a 0.45 μm cellulose acetate membrane to collect the brown-red solids. Then, the collected solids were rinsed alternately several times using ethanol and ultrapure water, and vacuum-dried at 60 °C overnight. Finally, the dried solid samples were scanned by transmission electron microscopy (TEM) to capture the microscale crystallization and structure and energy dispersive X-ray spectroscopy (EDX) to analyze the elemental composition.

## 3. Results and discussion

### 3.1. Feasibility of sludge dewatering with the KMnO_4_/PMS process

**Fig. 1:**
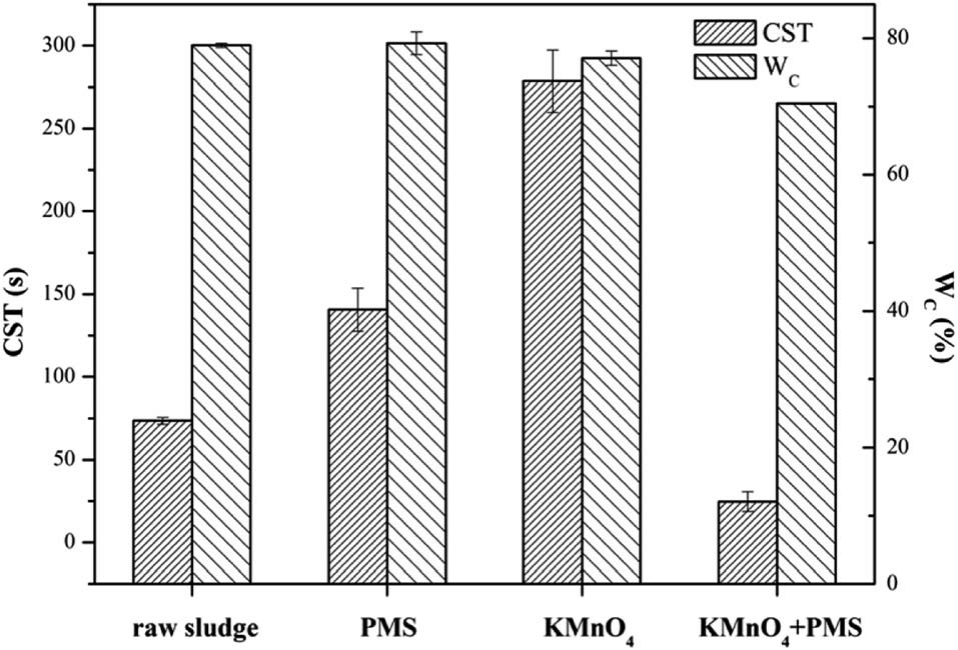
Dewaterability change in the WAS samples conditioned by different methods (experimental conditions: [KMnO_4_] = [PMS] = 7 mmol g^−1^ VSS, [KMnO_4_] + [PMS] = 4 mmol g^−1^ VSS + 3 mmol g^−1^ VSS, pH_0_ = 7, *T* = 25 °C).

Fig. 1 compared the dewaterability of the WAS samples conditioned by KMnO_4_ alone, PMS alone, and a combination of KMnO_4_ and PMS (KMnO_4_/PMS), respectively. As observed, the CST increased from 73.65 s to 140.60 s and 278.55 s, respectively when the WAS samples were conditioned by 7 mmol g^−1^ VSS PMS and KMnO_4_ alone, respectively, which indicated that neither PMS alone nor KMnO_4_ alone could efficiently improve the sludge dewaterability, and that the two individuals even worsened the dewaterability. The deterioration of sludge dewaterability was likely resulted from the progressive cell lysis and further releasing of intracellular biopolymers under the influence of KMnO_4_ or PMS when the intracellular biopolymers could not be degraded effectively in current condition.^33^ Lee *et al.* also found that the PMS conditioning could deteriorate the dewaterability after the WAS samples were treated with different dosages of PMS at room temperature.^5^ Surprisingly, a combined use of 4 mmol g^−1^ VSS KMnO_4_ and 3 mmol g^−1^ VSS PMS instantly decreased the CST from 73.65 s to 24.65 s, and apparently showed its synergistic effect on significantly improving the sludge dewaterability. Besides, the decrease of *W*_C_ (from 78.96% to 70.47%) also indicated the synergistic effect after the WAS samples were conditioned by the KMnO_4_/PMS process while the *W*_C_ values almost remained unchanged after the samples were treated with PMS alone and KMnO_4_ alone (79.25% and 77.07%, respectively). The synergistic effect of the KMnO_4_/PMS process on the WAS dewaterability was interpreted with the decrease of both CST and *W*_C_, which also implied the generation of SO_4_˙^−^ and HO˙ during the KMnO_4_/PMS process as mentioned in Section 1.

### 3.2. Effect of the KMnO_4_/PMS process on the physicochemical characteristics of the WAS samples

#### 3.2.1. Capillary suction time (CST)

**Fig. 2:**
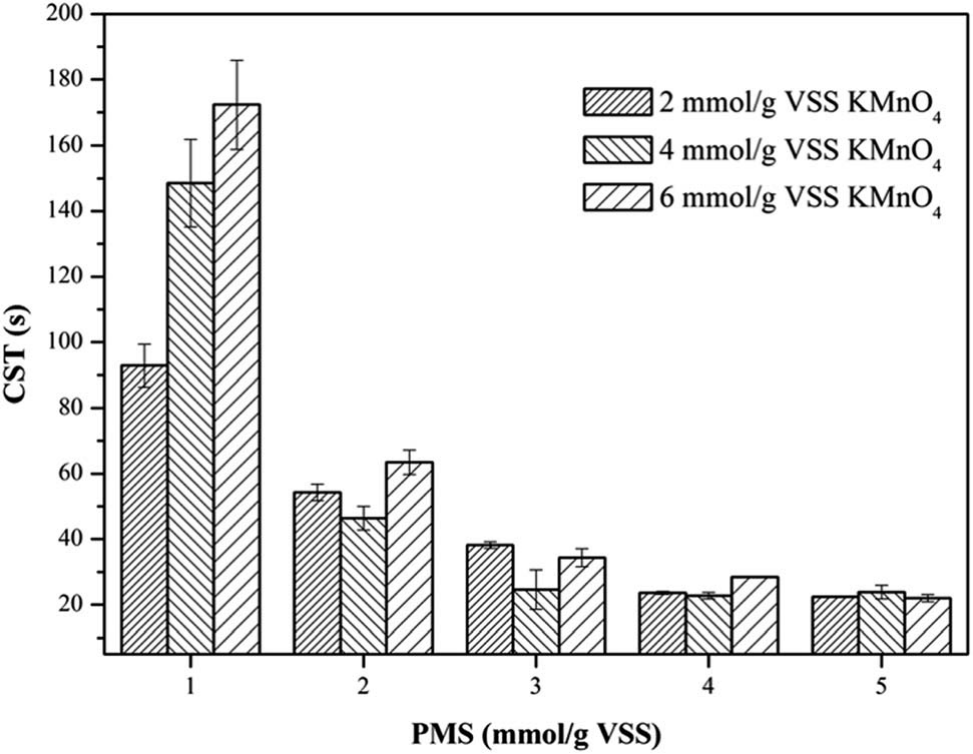
Effect of the KMnO_4_/PMS process on CST of the WAS samples (experimental conditions: [KMnO_4_] = 2–6 mmol g^−1^ VSS, [PMS] = 1–5 mmol g^−1^ VSS, pH_0_ = 7, *T* = 25 °C).

The dewaterability of the WAS samples were further investigated by simultaneously dosing different amounts of KMnO_4_ and PMS. As shown in Fig. 2, when PMS dosage was fixed at 1 mmol g^−1^ VSS, the increase of KMnO_4_ dosage from 2 mmol g^−1^ VSS to 6 mmol g^−1^ VSS did not decrease the CST, and instead jeopardized the dewaterability, which indicated the absence of the synergistic effect of the KMnO_4_/PMS process at a low PMS dosage. This might be because the *in situ* generated SO_4_˙^−^ and HO˙ during the KMnO_4_/PMS process was too few to improve the dewaterability of the WAS samples when PMS dosage was too low. However, increasing PMS dosage to 2 mmol g^−1^ VSS efficiently enhanced the dewaterability, and the CST decreased from 73.65 s to 54.30 s, 46.45 s and 63.45 s, respectively with increasing KMnO_4_ dosage from 2 mmol g^−1^ VSS to 6 mmol g^−1^ VSS. When PMS dosage continuously increased to 5 mmol g^−1^ VSS, the CST decreased to 22.45 s, 23.85 s and 22.00 s, respectively as KMnO_4_ dosage increased from 2 mmol g^−1^ VSS to 6 mmol g^−1^ VSS. This is because the higher PMS dosage, the more SO_4_˙^−^ and HO˙ generated to achieve better sludge dewaterability.^34^ The similar results were also reported by Liu *et al.* when the Fe^2+^-activated PMS was used for sludge conditioning.^4^ Remarkably, the CST values did not decrease all the way, and instead decreased firstly and then increased with sustainably increasing KMnO_4_ dosage, which might be ascribed to the consumption of the generated SO_4_˙^−^ and HO˙ by KMnO_4_ with a too high dosage.^35^ Liu *et al.* also observed the similar results when using the magnetic Fe_3_O_4_–MnO_2_ core–shell nanocomposites-activated PMS for 4-chlorophenol degradation.^36^

#### 3.2.2. Water content of dewatered sludge cake (*W*_C_)

**Fig. 3:**
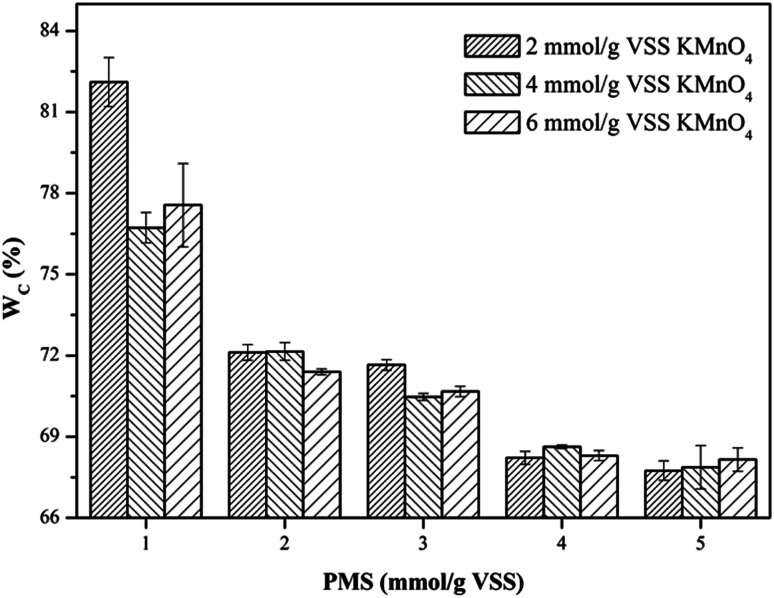
Effect of the KMnO_4_/PMS process on *W*_C_ of the WAS samples (experimental conditions: [KMnO_4_] = 2–6 mmol g^−1^ VSS, [PMS] = 1–5 mmol g^−1^ VSS, pH_0_ = 7, *T* = 25 °C).

The profiles of *W*_C_ of the WAS samples after conditioning of the KMnO_4_/PMS process with different KMnO_4_ and PMS dosages were depicted in Fig. 3. As can be seen, the *W*_C_ changes in the WAS samples were quite similar to the CST changes discussed in Section 3.2.1. By dosing 1 mmol g^−1^ VSS PMS, the *W*_C_ after conditioning with 2 mmol g^−1^ VSS, 4 mmol g^−1^ VSS and 6 mmol g^−1^ VSS KMnO_4_, respectively (82.11%, 76.73% and 77.56%) almost remained unchanged, compared to the *W*_C_ of the raw sludge (78.96%). This suggested that the synergistic effect of the KMnO_4_/PMS process with 1 mmol g^−1^ VSS PMS would not work. However, the *W*_C_ of the WAS samples would continue to decrease with the ever-increasing PMS dosages, which was also attributed to more SO_4_˙^−^ and HO˙ generated from the KMnO_4_/PMS process. The *W*_C_ after conditioning could decrease to about 67.5%, and by 11.46% compared to the *W*_C_ of the raw sludge, which was higher than the variation in the *W*_C_ of the WAS samples conditioned by the Fenton-like process (8.7%).^1^

#### 3.2.3. Zeta potential

**Fig. 4:**
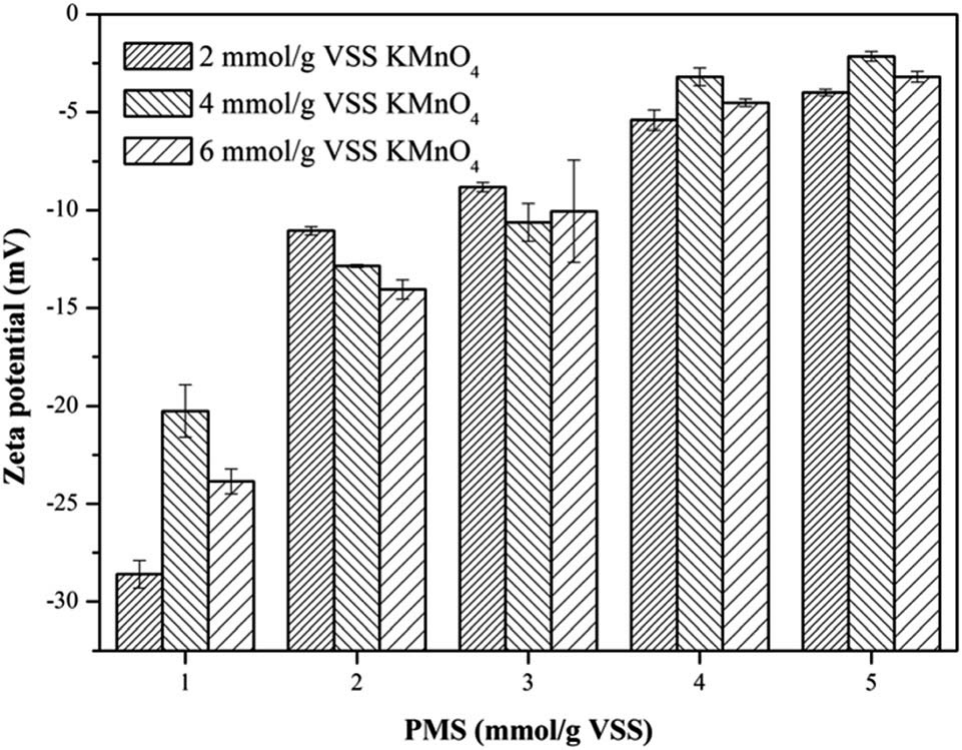
Effect of the KMnO_4_/PMS process on zeta potential of the WAS samples (experimental conditions: [KMnO_4_] = 2–6 mmol g^−1^ VSS, [PMS] = 1–5 mmol g^−1^ VSS, pH_0_ = 7, *T* = 25 °C).

To deeply investigate the effect of the KMnO_4_/PMS process on the WAS dewaterability, the zeta potentials of the WAS samples before and after conditioning were also measured. The unconditioned raw sludge was negatively charged with the zeta potential of −17.7 mV, which was due to the ionization of the carboxyl and amino in the protein of EPS.^37^ It can be obviously observed from Fig. 4 that the zeta potentials of the WAS samples were affected by dosing either KMnO_4_ or PMS. The zeta potential tended to increase to 0 mV with increasing KMnO_4_ or PMS dosage. By simultaneously dosing 4 mmol g^−1^ VSS KMnO_4_ and 5 mmol g^−1^ VSS PMS, the zeta potential increased to −2.15 mV. The increase in the zeta potential suggested the ever-increasing sludge dewaterability, which quite matched with the changes of the CST and *W*_C_. Zhen *et al.* also reported that the sludge dewaterability improved as the zeta potential increased in the Fe^2+^-activated persulfate process.^38^ According to the DLVO theory, the aggregation of sludge flocs is mainly controlled by the surface charge. The decrease in the negative surface charge of sludge flocs would reduce the electrostatic repulsion and bring about the increment of interaction energy, which led to the aggregation of sludge flocs and finally enhanced sludge dewaterability. Therefore, it can be concluded that sludge flocs are easier for agglomeration and sedimentation, and thus dewatering as the zeta potential increases.^39^

#### 3.2.4. Particle size distribution

**Fig. 5:**
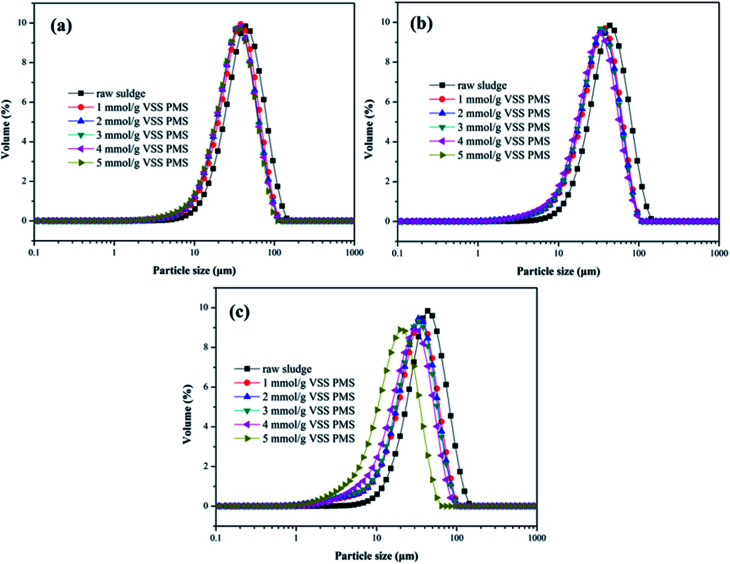
Effect of the KMnO_4_/PMS process on particle size distribution of the WAS samples: (a) 2 mmol g^−1^ VSS KMnO_4_; (b) 4 mmol g^−1^ VSS KMnO_4_; (c) 6 mmol g^−1^ VSS KMnO_4_ (experimental conditions: [PMS] = 1–5 mmol g^−1^ VSS, pH_0_ = 7, *T* = 25 °C).

As depicted in Fig. 5, effect of the KMnO_4_/PMS process on particle size distribution of the WAS samples was also investigated. The *D*_50_ value of the unconditioned sludge flocs was determined to be 39.2 μm. When the WAS samples were conditioned with 2 mmol g^−1^ VSS KMnO_4_ and 1 mmol g^−1^ VSS PMS, the *D*_50_ value decreased to 32.95 μm. With PMS dosage increasing to 5 mmol g^−1^ VSS, the *D*_50_ value also decreased to 30.08 μm. By dosing 4 mmol g^−1^ VSS and 6 mmol g^−1^ VSS KMnO_4_, the *D*_50_ values of the conditioned sludge flocs decreased from 30.42 μm and 28.35 μm to 25.78 μm and 24.44 μm, respectively with increasing PMS dosage. Therefore, increasing both KMnO_4_ and PMS dosages could reduce the size of the conditioned sludge flocs. The decrease of the sludge flocs size suggested that the KMnO_4_/PMS process could efficiently break the sludge flocs to enhance the sludge dewaterability as the flocs breakage could provide more passages for the removal of water in the inner of sludge flocs.^40^ It was also reported by Higgins and Novak that the sludge dewaterability was adversely impacted by the flocs in the range of 1–100 μm and decreased as the quantities of flocs in this size range increased.^41^

#### 3.2.5. Extracellular polymeric substances (EPS)

**Fig. 6:**
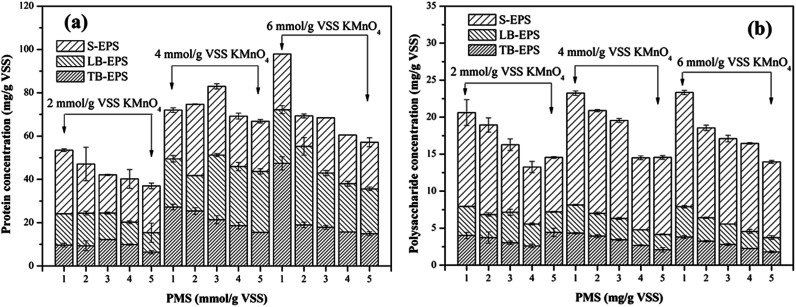
Effect of the KMnO_4_/PMS process on EPS of the WAS samples: (a) protein; (b) polysaccharide (experimental conditions: [KMnO_4_] = 2–6 mmol g^−1^ VSS, [PMS] = 1–5 mmol g^−1^ VSS, pH_0_ = 7, *T* = 25 °C).

EPS can have significant effects on the compressibility of sludge and the ability to bind water molecules of sludge flocs, and thus the dewaterability of sludge.^42^ Since EPS are mainly composed of protein and polysaccharide, variations in the concentrations of protein and polysaccharide in different EPS fractions (S-EPS, LB-EPS and TB-EPS) were monitored to reveal why the KMnO_4_/PMS process could improve sludge dewaterability. As illustrated in Fig. 6a, with the increase of PMS dosage, the protein content in total EPS tended to decrease. By dosing 2 mmol g^−1^ VSS KMnO_4_, the protein content in total EPS decreased from 53.46 mg g^−1^ VSS to 36.99 mg g^−1^ VSS while the protein content in S-EPS increased from 21.57 mg g^−1^ VSS to 29.31 mg g^−1^ VSS before decreasing to 21.59 mg g^−1^ VSS, as PMS dosage increased. Moreover, the protein content in LB-EPS decreased from 25.21 mg g^−1^ VSS to 9.01 mg g^−1^ VSS while the protein content in TB-EPS decreased from 9.68 mg g^−1^ VSS to 6.39 mg g^−1^ VSS. Similar changes of the protein content in total EPS, S-EPS, LB-EPS and TB-EPS could also be observed when 4 mmol g^−1^ VSS and 6 mmol g^−1^ VSS KMnO_4_ were dosed. In contrast to Fig. 6a and b showed that changes of the polysaccharide and protein content in EPS resembled each other.

The dissolved organics (protein and polysaccharide) in S-EPS increased before decreasing because TB-EPS were dissolved and transferred to S-EPS after the sludge flocs were destructed by SO_4_˙^−^ and HO˙ generated in the KMnO_4_/PMS process.^29^ Nevertheless, more SO_4_˙^−^ and HO˙ were generated to degrade the organics in S-EPS when PMS dosage continued to increase, so the protein and polysaccharide content in S-EPS tended to decrease. In addition, the slump of the protein and polysaccharide content in S-EPS, LB-EPS and TB-EPS accounted for SO_4_˙^−^ and HO˙ generation in the KMnO_4_/PMS process and its high reactivity toward S-EPS, LB-EPS, and even TB-EPS.^4^

### 3.3. Mechanism of WAS dewatering by the KMnO_4_/PMS process

Due to the versatility and efficiency, KMnO_4_ was deemed as an important precursor for the fabrication of manganese oxide nanomaterials through chemical reduction, including manganese dioxides, tetraoxides and oxyhydroxides.^43^ Wu *et al.* used KMnO_4_ as a conditioner for disintegration of excess activated sludge, and found that after sludge oxidation KMnO_4_ mainly transferred to the states of MnO_2_ and Mn_3_O_4_ while some compounds of K_*x*_MnO_4_ also existed in the solids.^28^ Due to the multivalent nature of Mn, MnO_2_ and Mn_3_O_4_ have been proven to possess a high catalytic activity toward PMS for SO_4_˙^−^ generation.^44,45^ Thus, in the KMnO_4_/PMS process, manganese oxides (MnO_*x*_) in the state of MnO_2_ and Mn_3_O_4_ might be generated from the reduction of KMnO_4_, then MnO_2_ and Mn_3_O_4_ further activated PMS to produce SO_4_˙^−^ and HO˙.

**Fig. 7:**
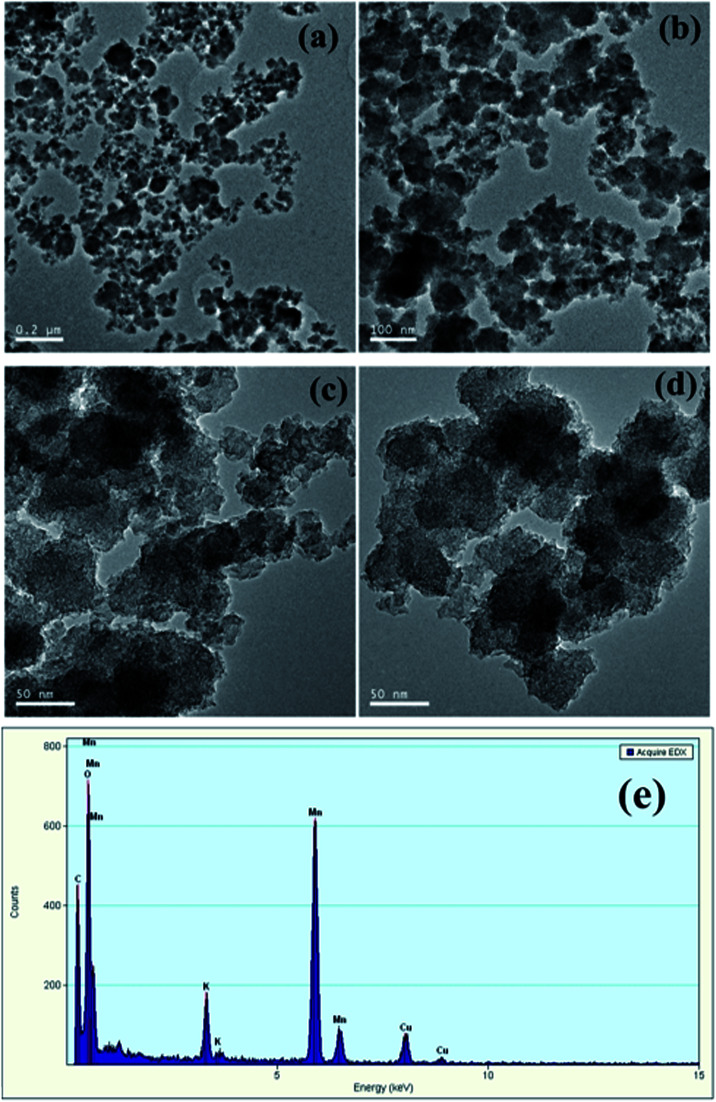
TEM image of the solid samples (a–d) and EDX spectra of the solid samples (e).

**Fig. 8:**
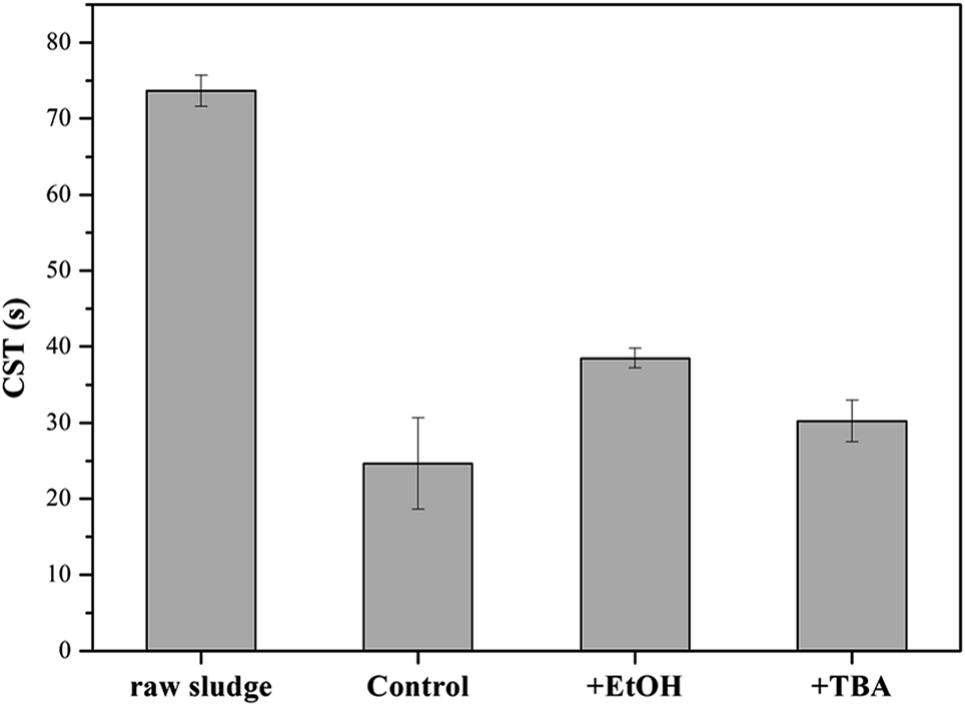
Effect of EtOH and TBA on the dewaterability of the WAS samples by the KMnO_4_/PMS process (experimental conditions: [KMnO_4_] + [PMS] = 4 mmol g^−1^ VSS + 3 mmol g^−1^ VSS, [EtOH] = [TBA] = 300 mmol g^−1^ VSS, pH_0_ = 7, *T* = 25 °C).

In order to verify the hypothesis, the formed solids were separated from the KMnO_4_/PMS process. And the samples were observed through TEM and EDX. From Fig. 7a–d, it could be clearly seen that some nanoscale solids did form in the KMnO_4_/PMS process. Moreover, the EDX analysis further confirmed that the formed solids were mainly composed of element Mn, O and K (Fig. 7e), and the weight percentages of Mn, O and K were recorded to be 20.9%, 74.26% and 4.82%. It suggested that KMnO_4_ was disintegrate into MnO_*x*_ under the influence of EPS. According to the previous study, the solids might also be MnO_*x*_ in the states of MnO_2_, Mn_3_O_4_ and K_*x*_MnO_4_.^28^ Meanwhile, scavenging experiments using ethanol (EtOH) and *tert*-butyl alcohol (TBA) as radical scavengers were conducted to confirm the formation of SO_4_˙^−^ and HO˙ in the KMnO_4_/PMS process. As can be seen in Fig. 8, after extra addition of 300 mmol g^−1^ VSS EtOH and TBA, the CST decreased from 73.65 s to 38.50 s and 30.25 s, respectively while decreasing from 73.65 to 24.65 s without addition of any radical scavenger. Moreover, the reduction value of CST with the addition of EtOH was higher than that with the addition of TBA. Such difference in CST value drop by the two radical scavengers implied that both SO_4_˙^−^ and HO˙ were generated in the KMnO_4_/PMS process because EtOH can effectively quench both SO_4_˙^−^ and HO˙ while TBA reacts much faster with HO˙ than SO_4_˙^−^.^25^

**Fig. 9:**
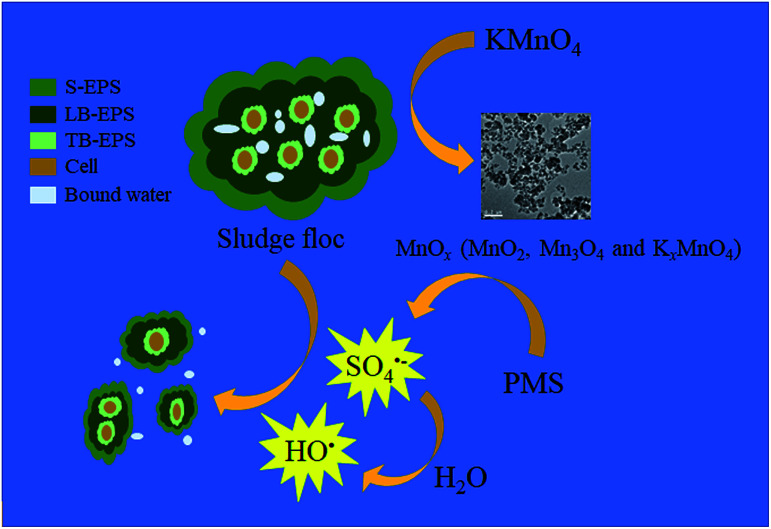
Schematic diagram of PMS activation by KMnO_4_ during the sludge dewatering process.

Based on both the results above and current studies, a possible mechanism was proposed. As depicted in Fig. 9, Firstly, KMnO_4_ transformed into MnO_2_, Mn_3_O_4_ and K_*x*_MnO_4_ after oxidizing the organics in EPS. Then, the *in situ* generated MnO_2_ and Mn_3_O_4_ efficiently activated PMS to generate SO_4_˙^−^ and HO˙ (eqn (2)–(5)). In detail, HSO_5_^−^ (the species of PMS) initially integrated with ≡Mn^IV^ and ≡Mn^III^ located at the surface of MnO_2_ and Mn_3_O_4_ by surface hydroxyl groups (eqn (6)). Then, ≡Mn^IV^ and ≡Mn^III^ would be reduced to ≡Mn^III^ and ≡Mn^II^, respectively with the generation of SO_5_˙^−^ (eqn (7)). Meanwhile, the formed ≡Mn^III^ and ≡Mn^II^ could be oxidized to ≡Mn^IV^ and ≡Mn^III^ with the formation of SO_4_˙^−^ (eqn (8) and (9)). Furthermore, HO˙ could also be produced through the reaction between SO_4_˙^−^ and H_2_O (eqn (5)).^46^ Finally, SO_4_˙^−^ and HO˙ degraded the organics in EPS to break the sludge flocs so that huge quantities of bound water were released and became free water, thus significantly enhancing sludge dewaterability.

2 2Mn_3_O_4_ + HSO_5_^−^ → 3Mn_2_O_3_ + SO_4_˙^−^ + H^+^

3 2MnO_2_ + HSO_5_^−^ → SO_5_˙^−^ + OH^−^ + Mn_2_O_3_

4 Mn_2_O_3_ + HSO_5_^−^ → SO_4_˙^−^ + 2MnO_2_ + H^+^

5 SO_4_˙^−^ + H_2_O → HO˙ + H^+^ + SO_4_^2−^

6 ≡Mn^IV(III)^ – OH + HSO_5_^−^ → ≡Mn^IV(III)^ − (O)OSO_3_^−^ + H_2_O

7 ≡Mn^IV(III)^ − (O)SO_3_^−^ + H_2_O → ≡Mn^III(II)^ − OH + SO_5_˙^−^ + H^+^

8 ≡Mn^III(II)^ − OH + HSO_5_^−^ → ≡Mn^III(II)^ − (O)OSO^−^_3_ + H_2_O

9 ≡Mn^III(II)^ − (O)OSO_3_^−^ + H_2_O → ≡Mn^IV(III)^ − OH + SO_4_˙^−^ + OH^−^

**Fig. 10:**
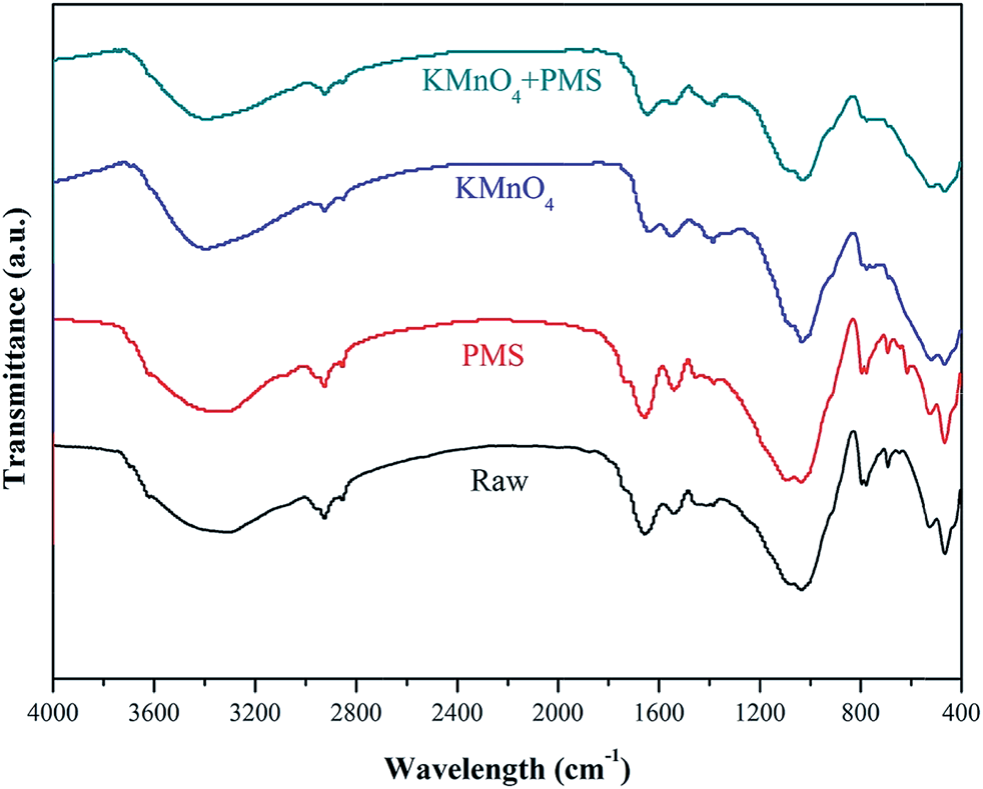
FTIR spectra of the WAS samples treated at different conditions.

FTIR spectrometer was also used to observed the variations of WAS functional groups after conditioning in different conditions. As illustrated in Fig. 10, the broad absorption band from 3200 to 3400 cm^−1^ could be attributed to the stretching vibrations of O–H group of polymeric substances.^47^ The peaks at 2925 and 2852 cm^−1^ were asymmetric and symmetric vibration of CH_2_ of aliphatic structures and lipids, respectively.^48^ The absorption band between 1640 to 1660 cm^−1^ was associated with the stretching vibrations of C=O and C–N, and C–N stretching vibration and N–H deformation vibration at 1550–1560 cm^−1^.^49^ The typical band located at 1040–1070 cm^−1^ was due to the stretching vibration of OH.^49^ Besides, some peaks (<1000 cm^−1^) monitored in the “fingerprint” region could be attributed to the phosphate or sulfur functional groups, which is the functional groups for the production of nucleic acids.^48^ Compared with conditioning by KMnO_4_ or PMS alone, it can be clearly seen that the intensity of absorption bands of WAS decreased obviously after treatment by the KMnO_4_/PMS process, implying that SO_4_˙^−^ and HO˙ generated in the KMnO_4_/PMS process could effectively degrade the proteins and polysaccharides in EPS and then resulted in the release of bound water and the improvement of sludge filterability.

## 4. Conclusion

This paper investigated the application of a combined use of KMnO_4_ and PMS (KMnO_4_/PMS) for sludge dewatering. The major conclusions can be drawn as followed:

(1) Compared with the separate use of KMnO_4_ or PMS, the combined use of KMnO_4_ and PMS (KMnO_4_/PMS) could significantly improve sludge dewaterability. After conditioning with the KMnO_4_/PMS process, CST of the WAS samples decreased from 73.65 s to 24.65 s. However, the dewaterability of WAS would be worsened by conditioning with KMnO_4_ alone or PMS alone because the CST value increased significantly.

(2) The KMnO_4_/PMS process exerted significant effects on the physicochemical characteristics of the WAS samples. When the WAS samples were treated by the KMnO_4_/PMS process, both CST and *W*_C_ would decrease, the zeta potential was less negative, and the particle size would also decrease as the sludge flocs were broken into smaller fragments. Besides, the concentrations of protein and polysaccharide in EPS were also reduced due to the synergistic effect of KMnO_4_ and PMS.

(3) The combined use of TEM and EDX techniques verified the generation of MnO_*x*_ in the states of MnO_2_, Mn_3_O_4_ and K_*x*_MnO_4_ after KMnO_4_ oxidation. The radical scavenging experiments confirmed that the *in situ* generated MnO_2_ and Mn_3_O_4_ could activate PMS to generate SO_4_˙^−^ and HO˙, which were responsible for significantly enhanced sludge dewaterability. Moreover, the FTIR analysis further indicated that SO_4_˙^−^ and HO˙ generated in the KMnO_4_/PMS process could improve sludge dewaterability.

## Conflicts of interest

There are no conflicts to declare.

## Supplementary Material
